# Characteristics of Early Antibody Mediated Rejection in Antibody Incompatible Living Donor Kidney Transplantation

**DOI:** 10.3389/ti.2024.12942

**Published:** 2024-07-08

**Authors:** Sai Rithin Punjala, Maria Ibrahim, Benedict Lyle Phillips, Jelena Stojanovic, Nicos Kessaris, Olivia Shaw, Anthony Dorling, Nizam Mamode

**Affiliations:** ^1^ Department of Transplantation, Guy’s and St Thomas’ NHS Foundation Trust, London, United Kingdom; ^2^ Department of Pediatric Nephrology and Transplantation, Great Ormond Street Hospital, London, United Kingdom; ^3^ Department of Pediatric Nephrology and Transplantation, Evelina Children’s Hospital, London, United Kingdom; ^4^ Clinical Transplantation Lab, Viapath, Guy’s and St Thomas’ NHS Foundation Trust, London, United Kingdom; ^5^ Department of Inflammation Biology, King’s College London, London, United Kingdom

**Keywords:** ABOi, HLAi, kidney transplantation, AMR, memory cells

## Abstract

Antibody incompatible transplantation (AIT) may be an only option for highly sensitized patients. Severe form of early antibody mediated rejection (AMR) adversely affects graft survival after AIT. The aim of this study was to identify individuals at risk of AMR. We analyzed 213 living donor AITs performed at our center. Among 120 ABOi, 58 HLAi and 35 DSA + FCXM-negative cases, the rates of early AMR were 6%, 31%, and 9%, respectively (*p* < 0.001). On multivariate analysis for graft loss, early AMR had a HR of 3.28 (*p* < 0.001). The HLAi group had worse death-censored graft survival (*p* = 0.003). In the HLAi group, Patients with aggressive variant AMR (AAMR) had greater percentage of C3d complement fixing DSA, higher baseline class I and total DSA MFI levels and B-cell FCXM RMF. C1q and C3d complement fixing DSA and strong positivity of baseline B- or T-cell FXCM as predictors of AAMR had 100% sensitivity. Early AMR is of significant clinical concern in AIT as it results in poor graft survival and is not well described in literature. An aggressive variant is characterized by massive rise in DSA levels at rejection. Baseline DSA, C1q, and C3d and baseline FCXM values can be used to risk-stratify candidates for AIT.

## Graphical Abstract

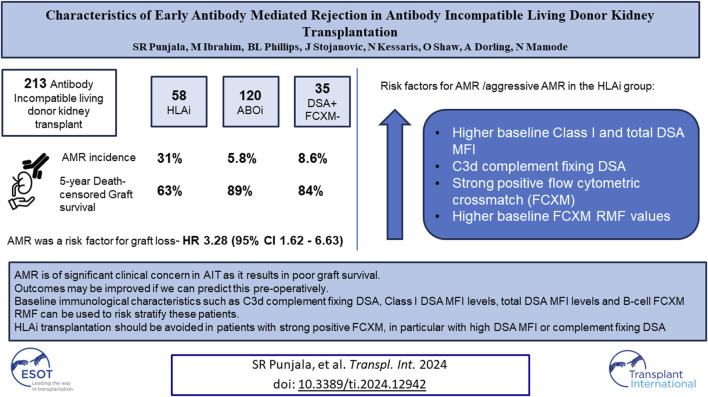

## Introduction

ABO-blood group incompatibility (ABOi) and Human Leukocyte antigen (HLA) sensitization have been barriers to direct kidney transplantation. The degree of sensitization is measured as calculated reaction frequency (cRF) in the UK [1], and as calculated panel reactive antibody (CPRA) in the US [2]. Kidney sharing schemes (KSS), prioritization of highly sensitized patients (HSP, cRF> 85% or CPRA> 80%) in national kidney allocation schemes, acceptable mismatch (AM) programs [3], and antibody incompatible kidney transplantation (AIT) have been successful in overcoming these barriers.

In the recent times, the number of annual kidney AITs has been in decline [4]. This can be attributed to the success of KSS which enable direct compatible transplantation. Although KSS have enabled a steady rise in the number of transplants performed every year, the number of transplants performed in individuals with a cRF 100% or CPRA 98%–100% have been very low [5, 6]. In the U.K, among the patients who wait for >7 years on the kidney only transplant waiting list, 98% are HSP [7]. Therefore, kidney allocation schemes have made provisions to prioritize HSP on the deceased donor waiting list to improve their transplant rates. Despite these provisions in the US, transplant rates remained extremely low in individuals with CPRA ≥99.9%. Any further modifications to the allocation policy may not improve the transplant rates [8]. Furthermore, allocation of organs with a poor HLA mismatch would increase the degree of sensitization in these recipients, rendering them more difficult to match for a future transplant.

Antibody mediated rejection (AMR) is now the most common cause of graft loss in kidney transplant recipients [9]. As anti-donor antibodies are responsible for AMR [10], the rates of AMR are higher in antibody incompatible kidney transplants compared to antibody compatible kidney transplantation [11]. AMR has been broadly classified based on the timing of rejection after transplantation, as early (<30 days) and late (>30 days). Early rejection usually occurs in individuals who undergo transplantation with preformed antibodies to donor antigens or in individuals who have an immunological anamnestic response from previous allo-sensitization [12].

Reports suggest that some patients suffering AMR within the first 2 weeks after transplantation are at particularly high risk of early graft loss [13, 14]. We hypothesize that this phenomenon, poorly described in literature, most likely occurs in patients with strong reactivation of their memory T and B cell responses, leading to a rapid increase in alloantibody production. The aim of this study was to identify, the incidence of AMR within the first 2 weeks after transplantation, and those at risk of early graft failure. Further, to better define the donor, recipient and baseline immunological characteristics associated with early AMR and its outcomes in antibody incompatible living donor kidney transplantation. This may help to risk stratify patients prior to transplantation.

## Materials and Methods

This study was a retrospective analysis of all antibody incompatible living donor kidney transplants performed at a UK Transplant center between 2005 and 2019. All blood group incompatible transplants with or without baseline DSA were grouped into ABOi transplants. All Flow Cytometry crossmatch (FCXM) positive transplants (relative mean fluorescence ratio, RMF >2.3) with or without blood group incompatibility were grouped into HLAi transplants, and all DSA positive but FCXM negative transplants (RMF <2.3) were grouped into “high-risk” transplants.

Our desensitization protocols for ABOi [15] and HLAi [16] kidney transplants have been published in the past. To summarize, we undertook antibody removal in ABOi patients with baseline titers of >64 with Glycosorb immunoadsorption (IA) columns, and in individuals with baseline titers between 16 and 64 we used double-filtration plasmapheresis (DFPP). No pre-operative antibody removal was performed in individuals with titers ≤8. In the HLAi group, antibody removal was carried out until a negative FCXM (RMF <2.3) was achieved. If multiple sessions of antibody removal were necessary to achieve a negative FCXM, immunoadsorption using Therasorb columns was preferred due to its minimal effect on coagulation. In all other cases, plasma exchange (PEX) or DFPP was used.

Our immunosuppression protocols have undergone modifications over the course of this study. In the ABOi transplants with no DSA, rituximab was given 4 weeks before a transplant at a dose of 375 mg/m^2^. In the initial period of this study, rituximab was given to all patients irrespective of their baseline ABO titers. This was later modified, and the new threshold for rituximab induction was set at ABO titers ≥8. All ABOi transplants received basiliximab induction on the day of transplant. Alemtuzumab induction was used in place of rituximab and basiliximab in ABOi transplants who have DSA. In the HLAi patients, basiliximab was used in the initial period of the study. Alemtuzumab induction has been used since July 2010. All HLAi transplants received low dose intravenous immunoglobulin (IVIg), at 0.5 gm/kg, 1 day before transplant following the last session of antibody removal, unless contraindicated. Patients in the “high-risk” group received alemtuzumab at induction from February 2011; prior to this, basiliximab induction was used. All patients in this study received standard triple maintenance immunosuppression (tacrolimus, mycophenolate mofetil and prednisolone). Participants of a randomized controlled trial looking into safety and efficacy of eculizumab in the prevention of AMR in antibody incompatible living donor kidney transplantation (NCT01399593) were included in this study. The impact of eculizumab on graft survival in multivariate analysis was not studied as some of the patients in the study received prophylactic eculizumab in the treatment arm.

Ethnic groups other than white were grouped together as ethnic minorities. Estimated glomerular filtration rate (eGFR) was calculated by using the Modification of Diet in Renal Disease equation [17]. MDRD eGFR was not collected in pediatric recipients as it is not an accurate marker of graft function in this population. RMF >2.3 but <2.8 were considered weak positive FCXM, and RMF >2.8 was considered as strong positive FCXM.

All FCXM negative, blood group incompatible transplants with or without DSA have been grouped into the ABOi group. We have defined HLAi as DSA positive and FCXM positive cases; and labelled all DSA positive but FCXM negative cases as “high-risk,” using this as a control group for comparison of outcomes. Moreover, all blood group incompatible cases who also had a positive crossmatch, are grouped into the HLAi group as these cases behave more like HLAi rather than an ABOi transplant.

The primary outcome of interest was AMR within the first 2 weeks after transplantation. Cases were identified based on for-cause biopsies or on clinical diagnosis. The secondary outcome of interest was to look at the impact of early AMR on patient and graft outcomes; and to predict recipient, donor and immunological factors associated with early AMR.

Statistical analyses were performed using IBM SPSS Statistics for Mac, Version 26.0. Armonk, NY: IBM Corp. Normality of the data was determined using Shapiro-Wilk test. Comparisons for continuous variables were performed with parametric (Student’s t-test, ANOVA) and non-parametric tests (Mann-Whitney, Kruskal-Wallis rank sums test), depending on distribution. Categorical variables were compared with Fisher’s exact test or χ^2^ test. We used Kaplan-Meier, and the log-rank test to compare death-censored graft and patient survival between groups. Risk associations were estimated with the use of multivariable Cox proportional-hazards models. Clinically important factors were tested and fit into a cox model. This study was conducted as an audit under the auspices of hospital audit committee and was exempt from institutional review board approval as it was a retrospective observational study. This study was conducted in accordance with the standards laid down by Declaration of Helsinki.

## Results

During this study period, a total of 213 antibody incompatible living donor kidney transplants were performed at our center. Of these, 120 were ABOi (111- DSA negative, 9- DSA positive), 58 were HLAi (50- HLAi, 8- combined HLAi and ABOi) and 35 were high-risk kidney transplants. Demographic data are shown in Table 1.

**TABLE 1:** Baseline demographics according to ABOi, HLAi and High-risk groups.

		Total (N = 213)	ABOi (N = 120)	HLAi (N = 58)	High-risk (N = 35)	*p*-value
Recipient age, years		45 (33–55)	47 (28–56)	43 (35–54)	50 (43–56)	0.60
Recipient gender, n (%)	Male	106 (50)	71 (59)	22 (38)	13 (37)	0.008
	Female	107 (50)	49 (41)	36 (62)	22 (63)	
Recipient ethnicity, n (%)	White	180 (84.5)	101 (84)	49 (84.5)	30 (86)	0.98
	Ethnic minorities	33 (15.5)	19 (16)	9 (15.5)	5 (14)	
Donor age, years		44 (36–53)	46 (38–53)	40 (30–46)	49 (37–57)	0.003*
Donor gender, n (%)	Male	100 (48)	54 (45)	32 (57)	14 (44)	0.28
	Female	108 (52)	66 (55)	24 (43)	18 (56)	
Donor ethnicity, n (%)	White	176 (86)	105 (87.5)	47 (85.5)	24 (80)	0.57
	Ethnic minorities	29 (14)	15 (12.5)	8 (14.5)	6 (20)	
Dialysis status pre-transplant, n (%)	Pre-emptive	53 (25)	41 (34)	3 (5)	9 (26)	<0.001
	HD	126 (59)	57 (48)	46 (79)	23 (66)
	PD	34 (16)	22 (18)	9 (16)	3 (8)	
Duration on dialysis, months		19 (0–51)	10 (0–31)	60 (29–129)	17 (2–43)	<0.001
Previous transplant, yes, n (%)		72 (34)	27 (37.5)	31 (43)	14 (19)	<0.001
Median peak cRF, %		28.5 (0–95)	0 (0–17)	98 (91–100)	87 (55–99)	<0.001^§^
Baseline, total DSA MFI			3,718 (2,210–4,710)	15,530 (9,630–25,849)	5,091 (3,300–8,777)	<0.001^ϕ^
Blood group incompatibilities, n (%)	A into O		62 (51.6)	4 (50)		
	B into O		19 (15.8)	2 (25)		
	AB into O		1 (0.8)			
	B into A		13 (10.8)	1 (12.5)		
	AB into A		8 (6.6)			
	A into B		14 (11.6)	1 (12.5)		
	AB into B		3 (2.5)			

Values expressed as Median (IQR), unless otherwise stated. Abbreviations: ABOi, ABO, blood group incompatible kidney transplantation; HLAi, Human Leukocyte antigen incompatible kidney transplantation; High-risk, DSA, positive and crossmatch negative transplant; IQR, inter quartile range; HD, hemodialysis; PD, peritoneal dialysis; cRF, calculated reaction frequency; DSA MFI, donor specific antibody median fluorescence index. *p = 0.007 and *p* = 0.016 when HLAi, group is compared to ABOi, and high-risk groups, respectively. ^§^ *p* < 0.001 when ABOi group is compared to HLAi and high-risk groups; *p* = 0.309 when HLAi, and high-risk groups were compared. ^ϕ^DSA, in ABOi, group included only nine patients who were DSA, positive.

A total of 29 patients were treated for AMR within the first 2 weeks after transplantation. After examining individual cases, one case was excluded from our analysis as review of allograft biopsy suggested recurrence of primary disease (Henoch-Schölen purpura) and no evidence of AMR (Supplementary Table S1), giving an overall early AMR rate of 13%.

Among the ABOi, HLAi and high-risk groups, the rates of AMR within 2 weeks were 5.8%, 31% and 8.6%, respectively (*p* < 0.001); the median days to AMR after transplantation were 6 (IQR, 6-7), 6.5 (IQR, 5–8.5) and 8 (IQR, 7–11), respectively (*p* = 0.447). The rates of graft survival at 1, 3 and 5 years were worse in the HLAi group, but there was no difference in 1, 3, and 5-year patient survival between the ABOi, HLAi and high-risk groups (Table 2).

**TABLE 2:** Rejection rates, graft and patient survival rates.

	ABOi (N = 120)	HLAi (N = 58)	High-risk (N = 35)	*p*-value
AMR, n (%)	7 (5.8)	18 (31)	3 (8.6)	<0.001
Median days to AMR, (IQR)	6 (6–7)	6.5 (5–8.5)	8 (7–11)	0.45
1-year DCGS	94% (88–97) n/N = 113/120	82% (69–90) n/N = 48/58	91% (75–97) n/N = 32/35	0.05
3-year DCGS	92% (85–96) n/N = 111/120	66% (51–77) n/N = 40/58	84% (66–93) n/N = 30/35	<0.001
5-year DCGS	89% (81–94) n/N = 108/120	63% (49–75) n/N = 39/58	84% (66–93) n/N = 30/35	<0.001
1-year patient survival	93% (87–97) n/N = 112/120	91% (80–96) n/N = 53/58	97% (81–100) n/N = 34/35	0.56
3-year patient survival	90% (83–94) n/N = 108/120	89% (77–95) n/N = 52/58	97% (81–100) n/N = 34/35	0.42
5-year patient survival	88% (80–93) n/N = 106/120	81% (67–89) n/N = 48/58	93% (73–98) n/N = 33/35	0.29

Graft survival and patient survival rates are expressed as mean survival rates (95% confidence interval). Abbreviations: ABOi, ABO, blood group incompatible kidney transplantation; HLAi, Human Leukocyte antigen incompatible kidney transplantation; high-risk, DSA, positive and crossmatch negative transplant; IQR, inter quartile range; DCGS, death-censored graft survival.

**FIGURE 1:**
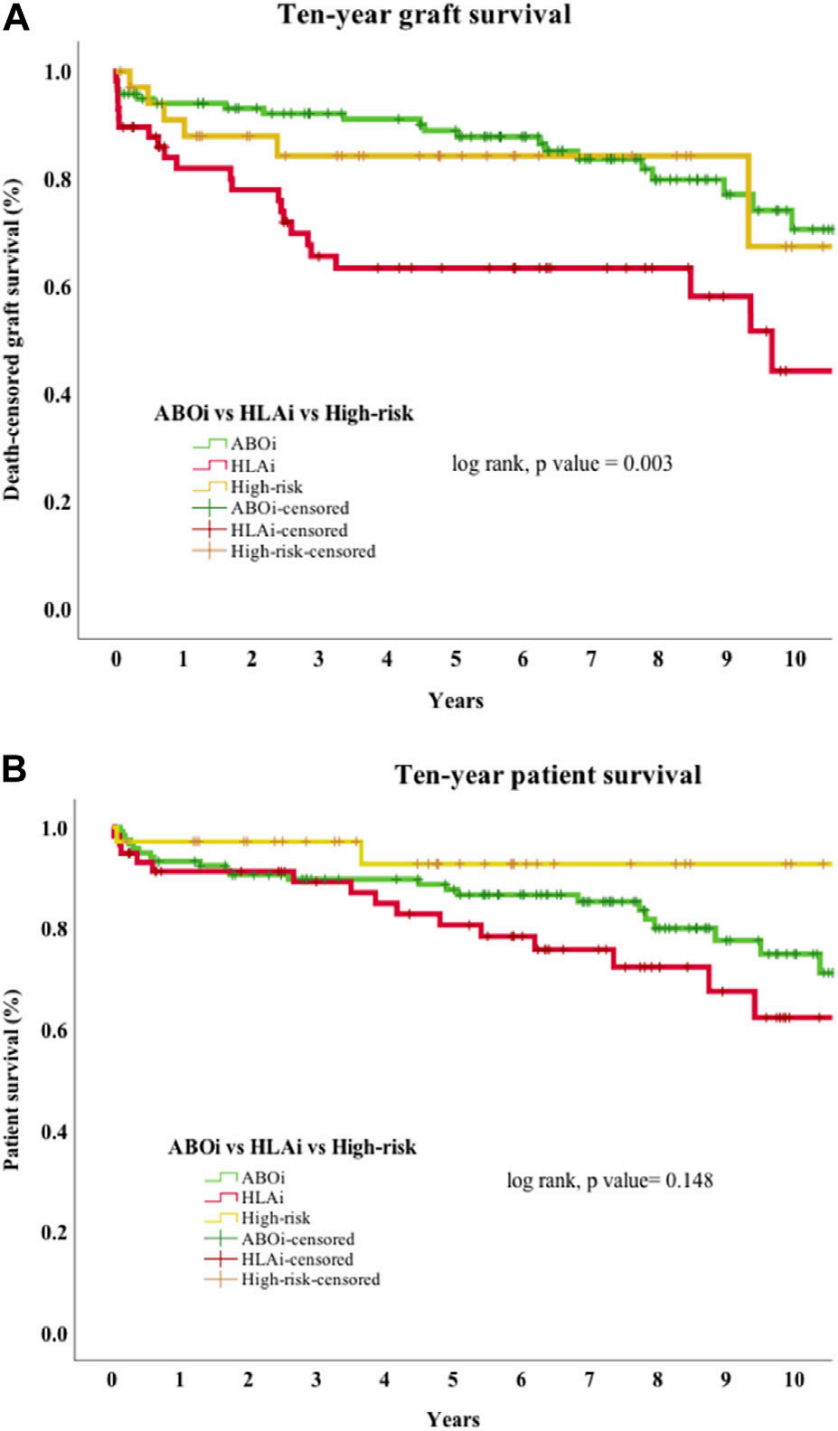
**(A)** Death-censored graft survival. Ten-year death-censored graft survival of HLAi group was worse compared to the ABOi and high-risk kidney transplant groups. Abbreviations: ABOi, ABO blood group incompatible kidney transplantation; HLAi, Human Leukocyte antigen incompatible kidney transplantation; high-risk, DSA positive and crossmatch negative transplant. **(B)** Patient survival. Ten-year patient survival of ABOi, HLAi and high-risk kidney transplants shows no difference in survival.

Early mortality (patient death <90 days from transplantation) was observed in 7 cases, and sepsis was the most common cause (*n* = 4/7, 57%). Incidentally, we noticed sudden unexpected death in 3 cases, all of whom received eculizumab. On univariate analysis, 30-day death-censored graft survival (DCGS) was worse in patients with AMR (97% vs. 75%, log rank *p* = <0.001). 10-year DCGS was inferior in the HLAi group compared to ABOi and high-risk groups (*p* = 0.003) (Figure 1A). There was no difference in the 10-year patient survival between the groups (*p* = 0.148) (Figure 1B). On the multivariable Cox regression modelling, AMR (hazard ratio (HR) 3.28; 95% confidence interval (CI) 1.62 to 6.63, *p* = <0.001) and second or more kidney transplant status (HR, 2.49; 95% CI 1.40 to 4.44; *p* = 0.002) were associated with overall DCGS. The following independent predictors of patient survival were identified: older recipient age (HR, 1.05; 95% CI 1.02 to 1.08; *p* < 0.001), longer duration spent on dialysis prior to transplant (HR, 1.009; 95% CI 1.002 to 1.01; *p* = 0.01) and eculizumab use for AMR treatment (HR, 2.77; 95% CI 1.01to 7.54; *p* = 0.04) (Table 3).

**TABLE 3:** Multivariable analysis on Cox regression model.

Cox regression model	Variable	HR	95% CI	*p*-value
Death-censored graft survival	No rejection	reference		
	AMR	3.278	1.62 to 6.63	<0.001
	Recipient previously on dialysis, yes	1.33	0.55 to 3.22	0.52
	Duration on dialysis	1	0.99 to 1	0.28
	Previous transplant (yes)	2.49	1.40 to 4.44	0.002
	Donor age	1.00	0.98 to 1.02	0.66
	Recipient age	1.00	0.98 to 1.02	0.66
Patient survival	Recipient age	1.05	1.02 to 1.08	<0.001
	Recipient previously on dialysis, yes	0.88	0.37 to 2.08	0.77
	Previous transplant (yes)	0.84	0.35 to 1.99	0.69
	Recipient gender	1.07	0.55 to 2.09	0.83
	Duration on dialysis	1.009	1.002 to 1.01	0.01
	Donor age	0.99	0.96 to 1.01	0.57
	AMR	1.26	0.48 to 3.30	0.62
	No eculizumab use	reference		
	Eculizumab use	2.77	1.01 to 7.54	0.04

This analysis includes all patients in the study, from all three groups. Choice of risk factors studied in this model was limited by the small sample size and limited number of significant events; therefore, only clinically significant risk factors, which are independent to classification of three subgroups, were included in this analysis. Abbreviations: HR, hazard ratio; 95% CI, 95% confidence interval; AMR, antibody mediated rejection; HLA, human leukocyte antigen.

Twenty-eight cases of early AMR were compared to 185 cases without AMR to characterize which patients were at an increased risk of early AMR (Table 4). Individuals in the early AMR group, were more likely to have received their kidney from a male donor (67% vs. 45%, *p* = 0.04), been on dialysis prior to their transplant (93% vs. 72%, *p* = 0.03), were on dialysis for a longer duration of time (44 vs. 17 months, *p* = 0.002), had higher baseline class I DSA MFI levels (18,700 vs. 9,127, *p* = 0.005), had a high-risk relation with the donor (husband to wife or child to mother) (39% vs. 20%, *p* = 0.03) and a greater percentage had DSA to repeat mismatches (62% vs. 20%, *p* = <0.001).

**TABLE 4:** Comparison between cases with and without AMR in the whole cohort of patients.

		No AMR (n = 185)	AMR (n = 28)	*p*-value
Donor age, years		45 (36–55)	42 (30–46)	0.07
Donor gender, n (%) (missing cases = 5)	Male	82 (45)	18 (67)	0.04
	Female	99 (55)	9 (33)	
Donor ethnicity, n (%) (missing cases = 8)	White	156 (88)	20 (74)	0.07
	Ethnic minorities	22 (12)	7 (26)	
Recipient age, years		45 (35–55)	43 (31–48)	0.18
Recipient gender, n (%)	Male	97 (52)	9 (32)	0.06
	Female	88 (48)	19 (68)	
Recipient ethnicity, n (%)	White	160 (87)	20 (71)	0.05
	Ethnic minorities	25 (13)	8 (29)	
Recipient dialysis status, n (%)	Pre-emptive	51 (28)	2 (7)	0.03
	HD or PD	134 (72)	26 (93)	
Duration on dialysis, months		17 (0–47)	44 (12–104)	0.002
Peak cRF		97 (81–99)	98 (95–100)	0.15
Total DSA MFI, baseline		9,125 (5,355–15530)	18,700 (7,017–40007)	0.005
Number of previous transplants, n (%)	First transplant	123 (66)	18 (64)	0.83
	Second or more	62 (34)	10 (36)	
High risk relation between recipient and donor, n (%)	No	147 (80)	17 (61)	0.03
	Yes	38 (20)	11 (39)	
HLA mismatch level, n (%)	Level 0, 1 and 2	37 (20)	7 (25)	0.61
	Level 3 and 4	148 (80)	21 (75)	
DSA to repeat mismatch, n (%) (missing cases = 6)	No	145 (80)	10 (38)	<0.001
	Yes	36 (20)	16 (62)	

Values expressed as Median (IQR), unless otherwise stated. Abbreviations: HD, hemodialysis; PD, peritoneal dialysis; cRF, calculated reaction frequency; HLA, human leukocyte antigen; DSA MFI, Donor specific antibody-mean fluorescence index. *DSA MFI, and cRF, values are presented only for HLAi, and high-risk transplant.

Since the HLAi group had greater percentage of cases with AMR (n/N = 18/28, 64%) and had worse overall graft survival, subgroup analysis of the HLAi group was performed to characterize which patients were at an increased risk of early AMR. Donor and recipient characteristics were not statistically different in cases with AMR when compared to cases without AMR. However, we observed that cases with AMR had significantly higher baseline class I DSA MFI levels (15,272 vs. 9,422, *p* = 0.03), baseline total DSA MFI levels (24,448 vs. 13,814; *p* = 0.01), pre-transplant class I DSA MFI levels (10,286 vs. 3,459, *p* = 0.03), greater percentage of cases with pre-transplant strongly positive FCXM (47% vs. 14%, *p* = 0.01), higher baseline T-cell FCXM RMF (RMF 3.22 vs. 2.41, *p* = 0.047) and baseline B-cell FCXM RMF (RMF 5.69 vs. 3.70, *p* = 0.03) and strong positivity of baseline B or T cell FXCM as predictors of early AMR had sensitivity of 100% (Table 5). A cut-off value of baseline total DSA MFI of 24,000 as predictor of AMR in the HLAi group has a sensitivity and specificity of 50% and 85%, respectively (ROC AUC = 0.70).

**TABLE 5:** Subgroup analysis- Comparison between cases with and without AMR in the HLAi group.

		No AMR (n = 40)	AMR (n = 18)	*p*-value
Donor age, years		40 (27–47)	43 (30–46)	0.92
Donor gender, n (%) (missing cases n = 2)	Female	18 (46)	6 (35)	0.56
	Male	21 (54)	11 (65)	
Donor ethnicity, n (%) (missing cases n = 3)	White	34 (90)	13 (77)	0.23
	Ethnic minorities	4 (10)	4 (23)	
Recipient age, years		42 (35–54)	45 (39–54)	0.45
Recipient gender, n (%)	Female	22 (55)	14 (78)	0.14
	Male	18 (45)	4 (22)	
Recipient ethnicity, n (%)	White	36 (90)	13 (72)	0.12
	Ethnic minorities	4 (10)	5 (28)	
Recipient dialysis status, n (%)	Pre-emptive	3 (7)	0	0.54
	HD or PD	37 (93)	18 (100)	
Duration on dialysis, months		57 (26–131)	75 (41–128)	0.45
Previous transplant, n (%)	No	17 (42)	10 (56)	0.40
	Yes	23 (58)	8 (44)	
High risk relation*, n (%)		9 (23)	8 (44)	0.12
Peak cRF		97 (87–99)	98.5 (95–100)	0.12
HLA mismatch level, n (%)	Level 0, 1 and 2	6 (15)	1 (6)	0.41
	Level 3 and 4	34 (85)	17 (94)	
DSA to repeat mismatches, n (%) (missing cases n = 4)	No	17 (45)	4 (25)	0.23
	Yes	21 (55)	12 (75)	
C1q complement fixing DSA, n (%) (missing cases n = 20)	No	16 (55)	2 (22)	0.18
	Yes	13 (45)	7 (78)	
C3d complement fixing DSA, n (%) (missing cases n = 20)	No	19 (65)	2 (22)	0.05
	Yes	10 (35)	7 (78)	
Class I DSA MFI, baseline		9,422 (5,516–12831)	15,272 (9,976–24867)	0.03
Class II DSA MFI, baseline		3,138 (0–9,565)	10,301 (0–26489)	0.15
Total DSA MFI, baseline		13,814 (9,069–22320)	24,448 (14,735–42491)	0.01
Class I DSA MFI, pre-transplant (missing cases n = 13)		3,459 (1,426–7,478)	10,286 (2,620–13953)	0.03
Class II DSA MFI, pre-transplant (missing cases n = 13)		1,412 (0–5,159)	2,958 (0–8,516)	0.48
Total DSA MFI, pre-transplant (missing cases n = 5)		6,946 (3,538–10728)	11,686 (5,432–20283)	0.08
FCXM, B or T cell, baseline, n (%) (missing cases n = 1)	Negative^**µ**^	2 (5)	0	0.08
	Weak positive	7 (18)	0	
	Strong positive	30 (77)	18 (100)	
FCXM, B or T cell, pre-transplant, n (%) (missing cases n = 5)	Negative	24 (67)	9 (53)	0.01
	Weak positive	7 (19)	0	
	Strong positive	5 (14)	8 (47)	
Baseline T-cell FCXM, RMF		2.41 (1.52–3.36)	3.22 (2.12–6.23)	0.047
Baseline B-cell FCXM, RMF		3.70 (2.81–6.18)	5.69 (4.35–9.75)	0.03
Pre-transplant T-cell FCXM, RMF (only 1 case in each group)		N/A	N/A	
Pre-transplant B-cell FCXM, RMF (among positive cases)		2.7 (2.47–3.27)	3.85 (3.03–3.95)	0.04

Values expressed as Median (IQR), unless otherwise stated.

^**µ**^These two cases were grouped in HLAi, as their pre-transplant FCXM, was weakly positive, *High risk relation between donor and recipient includes child to mother, or husband to wife relationship. Abbreviations: HLAi, Human Leukocyte antigen incompatible kidney transplantation; AAMR, aggressive antibody mediated rejection; cRF, calculated reaction frequency; DSA, donor specific antibody; MFI, median fluorescence index; FCXM, flow cytometry crossmatch; RMF, relative mean fluorescence ratio.

There was no significant difference in the MDRD eGFR between ABOi, HLAi and high-risk groups at 1, 3, and 5-year post-transplant. There was also no observed difference in MDRD eGFR at 1, 3, and 5-year, among individuals with and without AMR.

**FIGURE 2:**
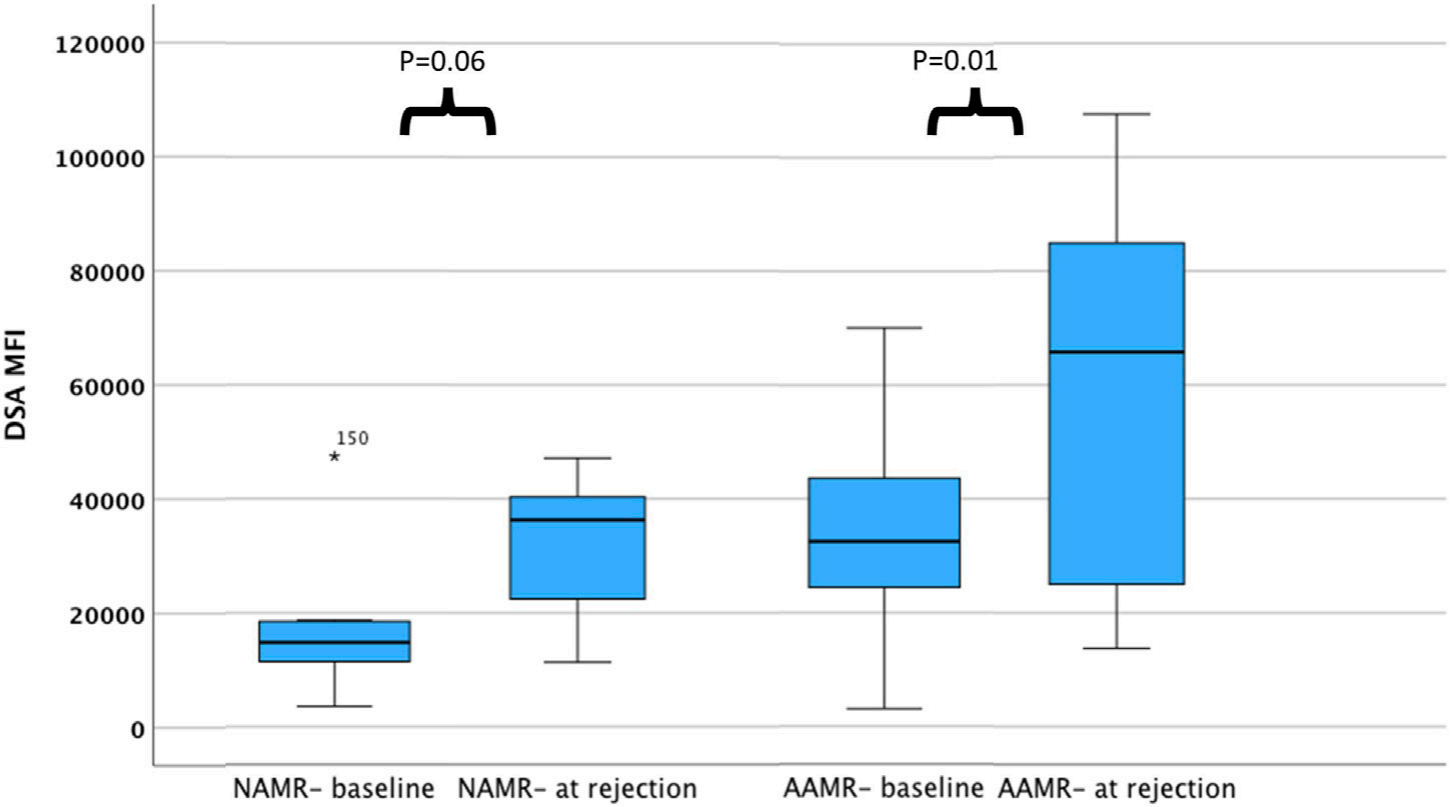
DSA levels among cases with AMR at baseline and at rejection. The bar chart illustrates DSA MFI levels among cases with AAMR and NAMR, at baseline and at rejection. Compared to baseline, DSA MFI levels are higher at rejection for cases with aggressive AMR. Abbreviations: DSA MFI: DSA, donor specific antibody median fluorescence index; AAMR, aggressive AMR; NAMR, non-aggressive AMR.

In an *ad hoc* analysis, we attempted to differentiate cases of AMR based on outcomes. Cases of AMR leading to graft loss, or not responding to initial therapy and subsequently needing eculizumab, or with thrombotic microangiopathy (TMA) on biopsy were grouped together as Aggressive AMR (AAMR). The rest of the cases were grouped together as non-aggressive AMR (NAMR) (Supplementary Table S1). Subgroup analysis was performed to characterize AAMR in HLAi group. In the AAMR group (n = 11), a massive increase in DSA MFI levels were observed at rejection when compared to baseline levels (DSA MFI 65797 vs. 32,519, *p* = 0.01). In the NAMR group (n = 7), no significant increase in DSA MFI levels were observed at rejection when compared to baseline levels (DSA MFI 36293 vs. 14,805, *p* = 0.06) (Figure 2). We observed that cases with AAMR had significantly higher baseline class I DSA MFI levels (17,872 vs. 9,422, *p* = 0.01), baseline total DSA MFI levels (32,519 vs. 14,583, *p* = 0.001), higher baseline B-cell FCXM RMF (RMF 6.44 vs. 3.86, *p* = 0.02) and pre-transplant B-cell FCXM RMF (RMF 3.93 vs. 2.86, *p* = 0.03) and a greater percentage had C3d complement fixing DSA (100% vs. 38%, *p* = 0.03). Strongly positive B/T cell FCXM, C1q and C3d complement fixing DSA as predictors of AAMR have a 100% sensitivity (Table 6). A cut-off value of baseline total DSA MFI of 23,000 as predictor of AAMR in the HLAi group had a sensitivity and specificity of 82% and 83%, respectively (ROC AUC = 0.81).

**TABLE 6:** Subgroup analysis- Comparison between cases with and without AAMR in the HLAi group.

		No AAMR (n = 47)	AAMR (n = 11)	*p*-value
Donor age, years		40 (31–46)	43 (27–51)	0.87
Donor gender, n (%) (missing cases n = 2)	Female	18 (42)	5 (45)	1.0
	Male	26 (58)	6 (55)	
Donor ethnicity, n (%) (missing cases n = 3)	White	37 (84)	10 (90)	1.0
	Ethnic minorities	7 (16)	1 (10)	
Recipient age, years		43 (35–54)	42 (35–56)	0.70
Recipient gender, n (%)	Female	28 (60)	8 (73)	0.50
	Male	19 (40)	3 (27)	
Recipient ethnicity, n (%)	White	39 (83)	10 (91)	1.0
	Ethnic minorities	8 (17)	1 (11)	
Recipient dialysis status, n (%)	Pre-emptive	3 (6)	0	1.0
	HD or PD	44 (94)	11 (100)	
Duration on dialysis, months		60 (31–133)	66 (16–96)	0.71
Previous transplant, n (%)	No	21 (45)	6 (55)	0.74
	Yes	26 (55)	5 (45)	
High risk relation*, yes, n (%)		12 (25)	5 (45)	0.27
Peak cRF		97 (89–99)	99 (95–100)	0.11
HLA mismatch level, n (%)	Level 0, 1 and 2	6 (13)	1 (9)	1.0
	Level 3 and 4	41 (87)	10 (91)	
DSA to repeat mismatches, n (%) (missing cases n = 4)	No	19 (43)	2 (20)	0.23
	Yes	25 (57)	8 (80)	
C1q complement fixing DSA, n (%) (missing cases n = 20)	No	18 (53)	0	0.11
	Yes	16 (47)	4 (100)	
C3d complement fixing DSA, n (%) (missing cases n = 20)	No	21 (62)	0	0.03
	Yes	13 (38)	4 (100)	
Class I DSA MFI, baseline		9,422 (5,516–13814)	17,872 (14,514–26958)	0.01
Class II DSA MFI, baseline		3,138 (0–9,565)	11,760 (773–26489)	0.12
Total DSA MFI, baseline		14,583 (9,037–21691)	32,519 (23,371–45928)	0.001
Class I DSA MFI, pre-transplant (missing cases n = 13)		3,663 (1,498–7,749)	11,686 (2,334–17249)	0.056
Class II DSA MFI, pre-transplant (missing cases n = 13)		1,299 (0–5,153)	5,770 (0–8,768)	0.33
Total DSA MFI, pre-transplant (missing cases n = 5)		7,080 (3,752–11359)	12,883 (4,034–23991)	0.10
FCXM, B or T cell, baseline, n (%) (missing cases n = 1)	Negative^**µ**^	2 (4)	0	0.43
	Weak positive	7 (15)	0	
	Strong positive	37 (81)	11 (100)	
FCXM, B or T cell, pre-transplant, n (%) (missing cases n = 5)	Negative	27 (64)	6 (54)	0.14
	Weak positive	7 (17)	0	
	Strong positive	8 (19)	5 (46)	
Baseline T-cell FCXM, RMF		2.5 (1.73–3.42)	3.42 (2.2–9.64)	0.07
Baseline B-cell FCXM, RMF		3.86 (2.82–6.3)	6.44 (5.34–10.10)	0.02
Pre-transplant T-cell FCXM, RMF (only 1 case in each group)		N/A	N/A	
Pre-transplant B-cell FCXM, RMF (among positive cases)		2.86 (2.49–3.3)	3.93 (3.65–4.08)	0.02

Values expressed as Median (IQR), unless otherwise stated. ^**µ**^These two cases were grouped in HLAi, as their pre-transplant FCXM, was weakly positive, *High risk relation between donor and recipient includes child to mother, or husband to wife relationship. Abbreviations: HLAi, Human Leukocyte antigen incompatible kidney transplantation; AAMR, aggressive antibody mediated rejection; cRF, calculated reaction frequency; DSA, donor specific antibody; MFI, median fluorescence index; FCXM, flow cytometry crossmatch; RMF, relative mean fluorescence ratio.

## Discussion

This study identifies that early AMR occurs more commonly in HLAi transplants as compared to ABOi and DSA positive FCXM negative transplants, at around 1-week post-transplant. An aggressive form of AMR (AAMR) (AMR leading to graft loss, or TMA on biopsy, or resistant to standard treatment) presents with massive antibody rise at rejection, far beyond baseline levels. It is likely a memory response, with B and T cell activation leading to increased antibody production. Patients with early AMR had higher baseline class I and total DSA, higher pre-transplant class I DSA, greater percentage of strongly positive pre-transplant FCXM, higher baseline T and B cell FCXM RMF, and strong positivity of baseline B or T cell FXCM as predictors of early AMR had sensitivity of 100%. Patients with aggressive AMR had a greater percentage of C3d complement fixing DSA, higher baseline Class I, total DSA MFI levels and B-cell FCXM RMF. Strongly positive B/T cell FCXM, C1q and C3d complement fixing DSA have 100% specificity as predictors of AAMR.

The main limitations of this study are the single center and retrospective observational nature of this study. Early AMR is relatively uncommon; given the low number of AMR events in the study population, interpretation of factors that may have influenced outcomes such as allograft loss, long-term allograft and patient survival may be difficult, as they can be influenced by various external factors. For the same reasons, direct comparisons between NAMR and AAMR were not made in the HLAi group. A low number of AMR events precluded us from performing a subgroup analysis in the ABOi and DSA positive FCXM negative groups. Analyses stratifying risk factors for AMR/AAMR in the HLAi subgroup are hampered by very low sample size, missing data and low number of significant events. This data should be interpreted with caution due to a high risk of both type I and type II errors. However, we do feel it is important to have these data in the manuscript, as the clinical implications of AMR/AMMR are quite devastating. A prospective national registry analysis may overcome some of these limitations. Further limitations of this study are the heterogeneity of the immunosuppression protocols and the treatment regimens used over time.

It is clear that ABOi and HLAi transplants are different entities, and rejection episodes in these groups should be usually discussed separately; ABOi transplants have a better graft survival and lower rejection rates. Risk aversion to unfavorable patient and graft outcomes have led to a decline in the number of AITs across different centers [18]. However, it is observed that the graft outcomes of HLA incompatible kidney transplants are comparable to compatible deceased donor kidney transplants [19]. Also, patient survival in individuals who undergo HLA incompatible living donor kidney transplantation is better than [20] or comparable to [21] individuals who wait on dialysis for a compatible transplant. In our study, the 5-year patient survival was 81%, which is much higher than the 5-year patient survival on dialysis. In individuals who are very highly sensitized (cPRA ≥98%) or unsuccessful in the kidney sharing schemes, antibody incompatible living donor kidney transplantation is sometimes the only option [22]. However, the long-term graft outcomes of antibody incompatible living donor kidney transplants are worse when compared to antibody compatible living donor kidney transplants [5]. This difference in long-term graft outcomes between ABOi and ABO-compatible living donor kidney transplants has been attributed to increased risk of graft loss within the first 14 days due to antibody mediated rejection (AMR) in the ABOi transplants [23]. In HLA incompatible (HLAi) living donor kidney transplants [24], this has been attributed to AMR occurring at different time periods [25] i.e., initial graft loss from early acute AMR (<30 days post-transplant) [14], and long-term graft loss from late acute AMR (>30 days post-transplant) [26, 27] or chronic AMR (CAMR) [28, 29].

Previous reports suggest accelerated acute rejection occurs around a week after transplant and represents an anamnestic response [13, 14]. Locke et al suggest early severe AMR after crossmatch positive live donor kidney transplant results in sudden onset oliguria/anuria with a rise in DSA; and may lead to graft loss if treated only with plasmapheresis and IVIg. In this series of five cases, splenectomy in addition to standard rescue therapy was able to salvage all kidney allografts [13]. Orandi et al report early severe AMR in 24 (9%) of their patients at a median of 6 days after HLA-incompatible living donor kidney transplantation. Sudden onset oliguria, rapid rise in serum creatinine and marked rebound of DSA were observed in these patients. This study reports 100% 1-year graft survival in patients treated with combined splenectomy and eculizumab, compared to 78% and 30% when treated with splenectomy alone and eculizumab alone, respectively. They suggest that while splenectomy debulks the active plasmablasts, high levels of antibodies still persist. Eculizumab, a monoclonal antibody that cleaves C5 complement, renders these antibodies ineffective, which may otherwise take days to be cleared by plasmapheresis [14].

We used eculizumab only in severe forms of rejection (AAMR) which were refractory to treatment with plasma exchange and IVIg. We feel that pre-emptive PEX in this group of patients is unlikely to make a difference, as the data that appears in this entity suggests that the antibody titers rise very quickly and manyfold. In our study, we observed that patients with AAMR had greater than two-fold increase in DSA MFI levels at rejection. Eculizumab is known to reduce the rates of early humoral rejection in sensitized individuals [30, 31], and has been recommended as adjunctive treatment therapy for early acute humoral rejection according to expert consensus from the transplantation society working group [12]. Of note, the use of eculizumab was associated with worse overall patient survival (Table 4). An important finding that needs to be further explored is the occurrence of sudden-onset early death (<90 days) due to a suspected cardiac cause in three individuals (11, 18, and 44 years old) who have been treated with eculizumab. These individuals have had no other identifiable cause of death. Eculizumab treatment should be initiated immediately after rejection, as its protective effect may not be durable if strong DSA is allowed to persist for long periods of time [32]; however, its risks and benefits should be assessed.

The long-term graft survival outcomes in our study were comparable to other large studies which looked at long term graft outcomes in ABOi [33] and HLAi kidney transplants [19]. Data suggests that C1q and C3d complement fixing DSA negatively affects long term graft survival [34, 35]. We looked at pre-transplant complement fixing DSA in only 38 (65%) patients in the HLAi group and found that patients with pre-transplant C3d complement fixing DSA were at an increased risk of AAMR. Also, patients with C1q and C3d complement fixing DSA had a sensitivity of 100% as predictors of AAMR. Massive increase of DSA MFI was observed in AAMR (N = 11) at rejection as compared to baseline (65,797 vs. 32,519, *p* = 0.01), whereas the non-aggressive AMR (N = 7) do not have a significant increase in MFI (36,293 vs. 14,805, *p* = 0.06). This data should be interpreted with caution. The DSA MFI approximately doubles in the two subcategories. What differentiates the two types of AMR is the higher baseline value in the aggressive AMRs. The borderline statistical significance is the result of the very low sample size of this subgroup analysis. In practice, we may avoid considering individuals with very high baseline total DSA MFI levels for an AIT as they may be at an increased risk of AAMR. We also advocate assessing complement data in more detail and avoid an AIT if the recipient has complement fixing DSA, pre-transplant.

We believe AAMR may be an anamnestic response from memory B and T-cells. One of the mechanisms of DSA formation is from interactions between CD4^+^ T-cells and donor HLA, via indirect pathway. Signals from these activated T-cells differentiates naïve B cells into antibody producing B-cells and antigen-specific memory B-cells. Memory B-cell survival is not dependent on continued exposure of antigen, and their threshold for activation is low. They rapidly expand and differentiate into short-lived antibody producing plasma cells on exposure to antigens [36]. Methods to identify HLA-specific memory B cells pre-operatively may risk-stratify candidates for AIT and prevent an anamnestic response [37]. Quantification of memory B-cells can be achieved using HLA specific B cell ELISpot assay. Interestingly, these donor specific memory B-cells may be present pre-transplant, despite the absence of circulating DSA [38]. Inflimidase is a protease that cleaves IgG antibodies. Our study identifies that high baseline DSA MFI levels, complement fixing DSA, and DSA against repeat mismatches with a previous failed transplant are risk factors for AAMR. Also, AAMR presents with significant rise in DSA MFI levels at rejection. The role of Inflimidase in desensitization protocols and treatment of AAMR needs to be further explored. Encouraging short-term graft and patient survivals were observed in HSP kidney transplant recipients with Inflimidase desensitization [39]. By routinely assessing memory B cells and Inflimidase use, we may be able to perform AIT with low short-term risk.

In conclusion, AAMR is of significant clinical concern in AIT as it results in poor graft survival and is not well described in literature. Outcomes may be improved if we can predict this pre-operatively. Baseline immunological characteristics such as C3d complement fixing DSA, Class I DSA MFI levels, total DSA MFI levels and B-cell FCXM RMF can be used to risk stratify these patients. HLAi transplantation should be avoided in patients with strong positive flow crossmatch, in particular with high DSA MFI or complement fixing DSA or DSA against repeat mismatches with a previous failed transplant. Complement inhibition can be successful if initiated early after rejection, but its use should be considered on an individual basis. AAMR may be due to T or B cell memory response, and methods to identify this pre-operatively would be an important area of future research.

## Data Availability

The raw data supporting the conclusions of this article will be made available by the authors, without undue reservation.

## Abbreviations

ABOi, ABO blood group incompatibility; aHUS, atypical hemolytic uremic syndrome; AIT, Antibody incompatible transplantation; AM, Acceptable mismatch program; AMR, Antibody mediated rejection; CDC, Complement-dependent cytotoxicity crossmatch; CPRA, Calculated panel reactive antibody; cRF, calculated reaction frequency; DCGS, Death-censored graft survival; DFPP, Double-filtration plasmapheresis; DSA, Donor specific antibody; EAAMR, Early Aggressive Antibody Mediated Rejection; FCXM, Flow cytometry crossmatch; HLAi, Human leukocyte antigen incompatibility; HSP, Highly sensitized patients; IA, Immunoadsorption; IVIg, Intravenous immunoglobulins; MFI, Mean fluorescence index; NAMR, Non-aggressive AMR; PEX, Plasma exchange; PNF, Primary non function; RMF, Relative mean fluorescence ratio; TMA, Thrombotic microangiopathy.
