# Supplementary material for: Characteristics of Early Antibody Mediated Rejection in Antibody Incompatible Living Donor Kidney Transplantation
Source: Transpl Int. 2024 Jul 8;37:12942. doi: 10.3389/ti.2024.12942 (PMC11261346; doi:10.3389/ti.2024.12942)
Supplement: Supplementary file 1 [file Table1.DOCX]

**Supplementary Table 1**. Immunological characteristics and allograft outcomes of patients with AMR.

| **Case ID** | **Transplant category** | **Baseline ABO titers** | **Baseline total DSA MFI** | **ABO titers at rejection** | **Total DSA MFI at rejection** | **Response to initial therapy** | **Eculizumab used for treating rejection due to failure of initial therapy** | **Biopsy result** | **TMA on biopsy** | **Rejection on post-operative day** | **Graft loss (days)** | **AAMR / NAMR** |
| --- | --- | --- | --- | --- | --- | --- | --- | --- | --- | --- | --- | --- |
| 41 | ABOi | 256 |  | 8 |  | No | No | glomerulitis, glomerular thrombi, fibrinoid necrosis of arteriolar wall, c4d3 | Yes | 3 | 6 | AAMR |
| 44 | ABOi +DSA | 4 | 3,718 | 8 | 12,942 | No | Yes | g0, ptc0, c4d3 | Yes | 7 |  | AAMR |
| 76 | ABOi | 4 |  | 256 |  | No | Yes | g1, ptc2, c4d3 | Yes | 7 | 15 | AAMR |
| 78 | ABOi | 64 |  | 8 |  | Yes | No | g3, ptc3, c4d1 | No | 5 |  | NAMR |
| 109 | ABOi | 2 |  |  |  |  | No | hyperacute rejection, g0, ptc2 | Yes | 0 | 0 | AAMR |
| 116 | ABOi +DSA | 128 | 4,710 | 1024 | 40,000 | No | Yes | g0, ptc1, c4d3 | Yes | 7 |  | AAMR |
| 120 | ABOi | 32 |  | 512 |  | No | Yes | g0, ptc0, c4d3 | No | 7 |  | AAMR |
| 131 | HLAi +ABOi | 16 | 3,093 | 32 | 13,750 | Yes | No | g0, ptc0, c4d3 | Yes | 6 | 1184 | AAMR |
| 132 | HLAi |  | 14,528 |  | 11,335 | Yes | No | g3, ptc3, c4d negative; cellular rejection IIA, t2, i2, v1 | No | 9 |  | NAMR |
| 134 | HLAi |  | 14,805 |  | 26,154 | Yes | No | g0, ptc1, c4d3 | No | 9 | 3531 | NAMR |
| 137 | HLAi |  | 18,319 |  | 47,173 | Yes | No | g3, ptc2, c4d2 | No | 9 | 226 | NAMR |
| 140 | HLAi |  | 38,669 |  | 17,174 | No | No |  | No | 8 | 16 | AAMR |
| 145 | HLAi |  | 23,371 |  | 52,419 | Yes | No | g0, ptc0, c4d3 | Yes | 8 |  | AAMR |
| 146 | HLAi +ABOi | 512 | 41,345 | 128 | 104,960 | Yes | No | g0, ptc0 | Yes | 4 |  | AAMR |
| 150 | HLAi |  | 47,453 |  | 42,467 | Yes | No | N/A (clinical diagnosis) | N/A | 10 | 177 | NAMR |
| 152 | HLAi |  | 27,103 |  | 27,582 | No | Yes | g1, ptc0, c4d3 | Yes | 5 |  | AAMR |
| 153 | HLAi +ABOi | 128 | 15,842 | 512 | 22,098 | No | No | day 5- g1, ptc1, c4d3.  day 7- Renal vein thrombosis. | Yes | 5 | 9 | AAMR |
| 159 | HLAi |  | 25,526 |  | 80,951 | No | Yes | N/A (clinical diagnosis) | N/A | 5 |  | AAMR |
| 164 | HLAi |  | 18,700 |  | 38,195 | No | No | g2, ptc2 | No | 5 | 1051 | NAMR |
| 170 | HLAi |  | 45,928 |  | 107,576 | No | Yes | N/A (clinical diagnosis) | N/A | 6 |  | AAMR |
| 171 | HLAi |  | 57,942 |  | 65,797 | No | Yes | g3, ptc1, c4d3 | Yes | 5 | 11 | AAMR |
| 172 | HLAi |  | 70,109 |  | 88,917 | No | Yes | N/A (clinical diagnosis) |  | 4 | 7 | AAMR |
| 174 | HLAi |  | 32,519 |  | 75,050 | No | Yes | g0, ptc0, c4d1 | Yes | 7 |  | AAMR |
| 175 | HLAi |  | 3,579 |  | 18,702 | Yes | No | N/A (clinical diagnosis) | N/A | 8 |  | NAMR |
| 177 | HLAi |  | 8,310 |  | 36,293 | Yes | No | N/A (clinical diagnosis) | N/A | 7 |  | NAMR |
| 180 | High risk |  | 3,300 |  | 4,900 | Yes | No | g1, ptc3 | No | 14 | 3404 | NAMR |
| 215 | High risk |  | 4,613 |  | 18,000 | Yes | No | g0, ptc0, c4d3, ATI | No | 8 |  | NAMR |
| 221 | High risk |  | 5,725 |  | 47,658 | Yes | No | g1, ptc1, ATI | No | 6 |  | NAMR |

Abbreviations: ABOi, ABO blood group incompatible kidney transplantation; HLAi, Human Leukocyte antigen incompatible kidney transplantation; NAMR, non-aggressive AMR; AAMR, aggressive AMR; DSA, donor specific antibody; MFI, median fluorescence index; TMA, Thrombotic microangiopathy. Criteria met for EAAMR in each case have been identified by bold font and underlined.

**Capsule sentence summary:**

Early AMR after AIT results in poor graft survival. HLAi transplantation should be avoided in patients with strong positive flow crossmatch, in particular with high DSA-MFI or complement fixing DSA or DSA against repeat mismatches with a previous failed transplant.
